# BdorOBP83a-2 Mediates Responses of the Oriental Fruit Fly to Semiochemicals

**DOI:** 10.3389/fphys.2016.00452

**Published:** 2016-10-05

**Authors:** Zhongzhen Wu, Jintian Lin, He Zhang, Xinnian Zeng

**Affiliations:** ^1^Key Laboratory of Natural Pesticide and Chemical Biology of the Ministry of Education, College of Natural Resources and Environment, South China Agricultural UniversityGuangzhou, China; ^2^Institute for Management of Invasive Alien Species, Zhongkai University of Agriculture and EngineeringGuangzhou, China

**Keywords:** *Bactrocera dorsalis*, olfactory, odorant binding proteins, functional analysis, attractive semiochemicals

## Abstract

The oriental fruit fly, *Bactrocera dorsalis* (Diptera: Tephritidae), is one of the most destructive pests throughout tropical and subtropical regions in Asia. This insect displays remarkable changes during different developmental phases in olfactory behavior between sexually immature and mated adults. The olfactory behavioral changes provide clues to examine physiological and molecular bases of olfactory perception in this insect. We comparatively analyzed behavioral and neuronal responses of *B*. *dorsalis* adults to attractant semiochemicals, and the expression profiles of antenna chemosensory genes. We found that some odorant-binding proteins (OBPs) were upregulated in mated adults in association with their behavioral and neuronal responses. Ligand-binding assays further showed that one of OBP83a orthologs, BdorOBP83a-2, binds with high affinity to attractant semiochemicals. Functional analyses confirmed that the reduction in BdorOBP83a-2 transcript abundance led to a decrease in neuronal and behavioral responses to selected attractants. This study suggests that BdorOBP83a-2 mediates behavioral responses to attractant semiochemicals and could be a potential efficient target for pest control.

## Introduction

In insects, olfaction plays a key role in behavior such as host-seeking, mating, and oviposition. At the molecular level, soluble binding proteins and membrane-bound receptors have crucial functions in chemical signal transduction (Pelosi et al., 2006, 2014; Zhou, 2010; Leal, 2013; Oppenheim et al., 2015). Odorant molecules penetrate into the sensilla via pore tubules and diffuse through sensillar lymph to membrane-bound receptor proteins. Activation of these proteins generates action potentials in receptor neurons. Two families of soluble binding proteins, odorant binding proteins (OBPs; Pelosi and Maida, 1995; Zhou, 2010) and chemosensory proteins (CSPs; Pelosi et al., 2005, 2006), are involved in this process.

Various functional studies support a role of insect OBPs in olfactory perception. Expression of moth pheromone receptors in heterologous systems (Grosse-Wilde et al., 2006, 2007; Forstner et al., 2009; Chang et al., 2015) and studies *in vivo* using the *Drosophila melanogaster* “empty neuron system” (Hallem et al., 2004; Syed et al., 2006) have demonstrated that the presence of a corresponding pheromone-binding protein significantly enhances the sensitivity of insects to pheromones. The OBP Lush with a mutation led to reduction in sensitivity of olfactory receptor neuron reception to the pheromone 11-cis-vaccenylacetate in *Drosophila* (Xu et al., 2005; Laughlin et al., 2008). RNA interference (RNAi) knockdown of OBPs lead to altered olfactory behavior in *Drosophila* (Swarup et al., 2011) and decreased electrophysiological responses in mosquito antennae (Biessmann et al., 2010; Pelletier et al., 2010a,b). There are two different hypotheses regarding the mechanisms of odor receptor (OR) activation. In moths and mosquitoes, OBPs act as solubilizers and carriers for the release of ligands onto ORs, thus contributing to the sensitivity of the insect olfactory system (Syed et al., 2006; Biessmann et al., 2010; Pelletier et al., 2010a). In *D. melanogaster*, LUSH (DmelOBP76a) forms an OBP-odorant complex that activates an OR, which is required for olfaction (Xu et al., 2005; Laughlin et al., 2008).

Insect CSPs are highly expressed in the sensillar lymph and exhibit binding activity toward odorants and pheromones (Jacquin-Joly et al., 2001; Gu et al., 2012; Iovinella et al., 2013; Zhang et al., 2014). CSPs of *Locusta migratoria* in antennae are involved in the physiological shift from solitary to gregarious phase (Guo et al., 2011), and an antenna-specific CSP of *Glossina morsitans morsitans* is thought to be associated with host searching behavior (Liu et al., 2012). However, there is no direct evidence to confirm a role of CSPs in olfaction.

The oriental fruit fly, *Bactrocera dorsalis* (Diptera: Tephritidae), is one of the most important pests throughout tropical and subtropical regions in Asia (Drew and Hancock, 1994). This insect pest can damage over 117 species of fruits and vegetables (Allwood et al., 1999), suggesting that it can detect and recognize a wide range of odorants. *B. dorsalis* exhibits remarkable developmental phases in olfactory behavior. When male adults reach sexual maturity, they are strongly attracted to and compulsively feed on methyl eugenol (4-allyl-1,2-dimethoxybenzene; Tan and Nishida, 2012). During the oviposition period, gravid females become more sensitive to a wide variety of volatile compounds, which have been shown to attract and/or stimulate oviposition (Jayanthi et al., 2014). These changes in olfactory behavior provide clues to study the physiological and molecular bases of olfactory perception in this pest, and may offer the possibility to explore alternative methods for pest control. Our previous transcriptome analysis of *B. dorsalis* provided a set of chemosensory genes and their expression profiles (Wu et al., 2015). Those antenna-specific or antenna-predominant chemosensory genes could be involved in recognition of specific ligands and contribute to olfactory behavioral changes in *B. dorsalis*.

The objective of the present study is to determine if there is a correlation between olfactory behavioral changes and those OBPs that are specifically or predominantly expressed in antennae. Specifically, we conducted behavioral assays, analyzed electroantennogram (EAG) responses to selected plant volatiles, and examined expression profiles of antenna-specific or antenna-predominant chemosensory genes in sexually immature and mated *B. dorsalis* adults. Using fluorescence competitive binding assays and molecular docking (*in silico*), binding affinity of selected OBPs to selected semiochemicals was also measured. The impact of the most abundant OBP, BdorOBP83a-2, on olfactory behavior was studied through RNAi. Our results suggest a likely involvement of antenna-specific or antenna-predominant OBPs in olfaction, especially in regulating foraging and oviposition behaviors.

## Materials and methods

### Insect rearing and collection

*B. dorsalis* used in this study was reared at the Institute for Management of Invasive Alien Species, Zhongkai University of Agriculture and Engineering, Guangzhou, China. Insects were reared under a photoperiod of 14:10 h (L:D) at 28°C and 70% relative humidity (Jayanthi and Abraham, 2002).

Sexually immature individuals (2 days old) and mated individuals (mated males and gravid females of 15 days old) were collected and used for the analyses of olfactory behavioral changes, olfactory sensitivity changes, and transcriptional changes of olfactory genes that are potentially induced by mating and gravidity. To obtain mated males and gravid females, 9 days old virgin adults of each sex were introduced into a 30 × 30 × 30 cm cage for mating. Once copulating pairs were formed for at least 60 min, they were transferred to another cage and maintained there until 15 days old.

### Semiochemicals

All semiochemicals used to investigate olfactory behavioral responses, EAG responses, and binding assays were purchased from Sigma-Aldrich (St. Louis, MO, USA) and with more than 95% purity (Table S1).

### Olfactory behavioral assays

Olfactory attraction was tested using a modified two-choice trap system based on the body size of *B. dorsalis* (Figure S1; Faucher et al., 2013). Odor traps were constructed from 250 ml conical flasks to which a silicone top containing 15 ml centrifuge tube (cut for 4.5 cm) was securely placed. Each trap contained a cotton-foam plug to which either 0.2 ml of 1% semiochemicals dissolved in paraffin oil or paraffin oil alone were added. Olfactory behavioral assays were conducted for 6 h in a dark humidified room at 28°C. Testing insects were starved for 4 h (6:00–10:00) in insect rearing cages with only water. Thirty insects per assay were then transferred to a new rearing cage. A performance index (PI) was used to measure olfactory attraction, which was calculated using the following formula: Performance index (PI) = (flies in odor vial – flies in control vial)/total of flies. Each trap assay was replicated five times.

### Electroantennogram (EAG) recording

EAG recordings (Syntech, Kirchzarten, Germany) were used to investigate *B. dorsalis* antennal responses to different semiochemicals according to standard protocols (Hayase et al., 2009). Insects were again starved for 4 h (6:00–10:00) before assays. The whole antennae of males and females were excised, cut on both ends and attached to two electrodes with Spectra 360 conducting gel (Parker Lab., Inc., Hellendoorn, Netherlands). Pure chemicals were dissolved in paraffin oil and tested at a final concentration of 10^−2^ v/v. A 10 μl aliquot of paraffin oil was used as a control. The neuronal responses of antennae from five males and five females were tested in each treatment. Five stimulations of each chemical were applied at intervals of 10 s on the antennae. EAG signals were amplified, filtered, digitized, and analyzed with an EAG Pro program (Syntech).

### Olfactory gene expression analysis

qRT-PCR was used to investigate the variation of the transcription levels of olfactory genes in sexually immature and mated adults. From each experiment, 100 whole antennae for each gender were excised and immediately transferred to a polypropylene tube cooled over liquid nitrogen. The frozen antennae were crushed, and total RNA was extracted with an RNeasy Mini kit (Qiagen), following the manufacturer's protocol. cDNA was synthesized from total RNA using a First strand cDNA synthesis kit (Takara). Specific primer pairs for the qRT-PCR were the same to those used in our previous study (Wu et al., 2015). The α-tublin (α-TUB; GenBank Acc. GU269902) of *B. dorsalis* was used for normalizing the target gene expression (Shen et al., 2010). qRT-PCR analysis was conducted on a LightCycler 480 system (Roche) with a SYBR Premix ExTaq kit (Takara). Negative controls without template were included in each experiment. To ensure reproducibility, each qRT-PCR reaction was performed using technical triplicates and biological triplicates. Relative gene expression data was estimated using the 2^−ΔΔCT^ method (Livak and Schmittgen, 2001).

### Bacterial expression and purification of BdorOBPs and BdorCSP

The sequences of BdorOBPs and BdorCSPs encoding mature proteins (without signal peptide) were amplified by PCR using specific primers carrying a restriction site EcoRI or XhoI (Table S2). PCR fragments were digested with both EcoRI and XhoI enzymes and the released DNA fragments were cloned into a PET-32a vector (Invitrogen), which were used to transform BL21 (DE3) *E. coli* competent cells (Invitrogen). A selected positive clone was grown overnight in 5 mL liquid LB/ampicillin medium overnight at 37°C. Protein expression was induced with 1 mM IPTG for 3 h when the culture had reached an OD_600_-value of 0.7–0.9. Cells were then harvested by centrifugation and lysed by sonication. After sonication and centrifugation, most recombinant proteins were present in inclusion bodies. Protein extracts were dissolved in extraction buffer (8 M urea, 0.5 M NaCl, 5 mM Imidazole, 1 mM β-mercaptoethanol, and 20 mM Tris-HCl pH 7.4) and purified using HisTrap affinity columns (GE Healthcare Biosciences, Uppsala, Sweden). Renaturation of purified proteins were accomplished by gradient dilution at 4°C. His-tag was removed by digestion with recombinant enterokinase (Novagen) and the digested mixtures were passed through a HisTrap affinity column to remove any undigested protein. At each step during protein preparation and purification, protein extracts, and purified proteins were examined on 15% SDS-PAGE.

### Fluorescence competitive binding assay

To measure the affinity of the recombinant proteins to the fluorescent probe N-phenyl-1-naphthylamine (1-NPN), a 2 μM solution of each target protein in 50 mM Tris-HCl buffer, pH 7.4, was titrated with aliquots of 1 mM 1-NPN in methanol to final concentrations of 1–16 μM. The affinity of the proteins to other ligands was measured in competitive binding assays, where a solution of the protein and 1-NPN, both at the concentration of 2 μM, was titrated with 1 mM methanol solution containing a competitor with concentrations in the range of 5–50 μM. Fluorescence spectra were measured on an F-7000 FL Fluorescence Spectrophotometer (Hitachi) in a 10 mm light path quartz cuvette at 25°C. All were excited at 337 nm with emission and excitation slit of both 10 nm. The emission spectra were recorded between 350 and 500 nm. Data analysis and plot binding curves were accomplished in the Prism software, assuming that the target protein had a 100% activity with a stoichiometry of 1:1 protein: ligand at saturation. All measurements were performed in triplicates and mean values and standard errors are calculated. The dissociation constants of the competitors were calculated from the corresponding IC_50_-values (the concentration of ligand halving the initial fluorescence value of 1-NPN), by the equation: K_D_ = [IC_50_]/1 + [1-NPN]/ K_1-*NPN*_, where [1-NPN] is the free concentration of 1-NPN and K_1-*NPN*_ is the dissociation constant of the complex Protein/1-NPN.

### Three-dimensional modeling and molecular docking

Two different strategies were employed to predict three-dimensional modeling of OBPs and CSP of *B. dorsalis*. Those that have more than 30% homology with the OBP or CSP templates in the Protein Database (http://www.rcsb.org/pdb) were predicted using the on-line program SWISS MODEL (Guex and Peitsch, 1997; Arnold et al., 2006; Guex et al., 2009), while others <30% were generated using an online protein threading (PHYRE 2; Kelley et al., 2015). The information of the templates was shown in Tables S3, S4. Alignments of BdorOBP83a-1, BdorOBP83a-2, BdorCSP3, BdorOBP84a-1, BdorOBP84a-2, and BdorOBP56h with the template protein are shown in Figure S2. Furthermore, rapid energy minimization of protein molecules was carried out using discrete molecular dynamics with an all-atom representation for each residue in the protein in Chiron (http://troll.med.unc.edu/chiron/login.php; Ramachandran et al., 2011). Molecular docking was performed using “Docking server” (Bikadi and Hazai, 2009). All compounds from previous studies were used for docking studies. Three-dimensional structures of compounds were obtained from NCBI. Docking was performed 255 times with selected OBPs and ligands. For each run, the 10 highest docking poses were saved and were further processed for molecular dynamics simulations. Free binding energies were calculated as previously described (Okimoto et al., 2009).

### Phylogenetic analysis and sequence alignment

Phylogenetic analyses of the newly identified *B. dorsalis* OBPs were performed in conjunction with previously identified *B. dorsalis* OBPs and OBPs from other species, including 31 OBPs from *B. dorsalis* (Wu et al., 2015), 52 OBPs from *D. melanogaster* (Hekmat-Scafe et al., 2002; Vieira et al., 2007; Vieira and Rozas, 2011), 16 OBPs from *Ceratitis capitata* (Siciliano et al., 2014a), 15 OBPs from *Rhagoletis pomonella* (Schwarz et al., 2009), 9 OBPs from *Rhagoletis suavis* (Ramsdell et al., 2010), Obp83a orthologs from *G. m. morsitans* (Liu et al., 2010; Macharia et al., 2016), *Musca domestica* (Scott et al., 2014), and *Calliphora stygia* (Leitch et al., 2015). After removal of signal peptide sequences, OBP amino acid sequences were aligned using MAFFT v6.935b (Katoh and Toh, 2010; Katoh and Standley, 2013) with the E-INS-i strategy, BLOSUM62 matrix, 1000 maxiterate and offset 0. The maximum-likelihood trees were constructed using FastTree 2.1.7 (Price et al., 2009, 2010) with 1000 bootstrap replications. The phylogenetic tree was visualized in FigTree (http://tree.bio.ed.ac.uk/software/figtree). For comparative purpose, the analyzed sequences were aligned using the E-INS-I strategy in MAFFT and visualized with Jalview 2.0.1 (Waterhouse et al., 2009).

### RNA interference and bioassay

dsRNAs for BdorOBP83a-2 and Bdorβ-gal (Glyceraldehyde-3-phosphate dehydrogenase) were synthesized through *in vitro* transcription from PCR products generated using a MEGAscript T7 transcription kit (Ambion). Briefly, the pMD18-T vector containing the target-gene insert (constructed as described above) was used as template for PCR amplification. PCR was performed using two specific primers with a T7 promoter sequence (5′-TAATACGACTCACTATAGGG-3′) at the 5′-end of each primer (Table S5). Large-scale dsRNAs were purified using a MEGAclear kit (Ambion) and precipitated with 5 M ammonium acetate to yield 30–50 μg/μL dsRNA. Ten days old adults were used for all injection experiments. Individuals receiving injection were placed on a stereomicroscope and surface-sterilized with 70% ethanol. One microgram of dsRNA was injected into each individual at the suture between third and fourth abdominal segments (100 ng/mg equivalent) using an automatic microinjection apparatus (Nanoject II Auto-Nanoliter). The injected insects were then transferred to new rearing cages for continuous culture (at 25 ± 1°C, 50% humidity) until 15 days old.

Given that rapid degradation of dsRNA fragments has been observed in insect hemolymph in other studies (Garbutt et al., 2013), we performed a pre-assay for the stability of dsRNA fragments under our conditions before starting dsRNA microinjection experiment. In the pre-assay, peripheral hemolymph was extracted from thoraxes of 10 days old adults individually using a microsyringe. Hemolymph from individual insects was combined into a precooled 50 μl PCR tube containing phenylthiourea to avoid melanization (Arakawa, 1995). The BdorOBP83a-2 dsRNA (1 μg) and hemolymph (3 μl) mixture was incubated at 25°C for 0–3 h. A control was included by mixing dsRNA with DEPC-water under the same conditions. After incubation, dsRNA was re-extracted with 20 μl nuclease-free water using an RNeasy Mini Kit (Qiagen, Valencia, CA) according to the RNA Cleanup instruction. Re-extracted dsRNA (8 μl) was separated on 1% agarose gels. Our *ex vivo* experiments showed that dsRNA fragments were stable in adult hemolymph, with a residence time of at least 3 h (Figure S3), indicating that dsRNA is likely to be stable in *B. dorsalis* for RNAi testing.

For PCR analysis, 30 pairs of antennae from 15 days old male and female adults, were excised and used for RNA isolation. Detailed procedures for RNA isolation, cDNA synthesis, and PCR amplification were the same as described previously. An α-tubulin (α-TUB; GenBank Acc. GU269902) of *B. dorsalis* was used as an internal reference. qRT-PCR primers used for BdorOBP83a-2 and Bdorβ-gal genes were designed with the Primer 3 program (http://primer3.ut.ee/) and the primer sequences are listed in Table S6. Technical triplicates and three biological replicates were carried out for each treatment.

EAG recordings were used to investigate any changes in antennal responses to attractant compounds (methyl eugenol and γ-octalactone) in water-treated, β-galactosidase-dsRNA-injected, BdorOBP83a-2-dsRNA-injected adults (both male and female). EAG responses were recorded for at least five individuals of water-treated control, β-galactosidase-dsRNA-injected control, and BdorOBP83a-2-dsRNA-injected insects. Each EAG observation was recorded with 10 individuals of each sex. Behavioral phenotypes of water-treated, β-galactosidase-dsRNA-injected, BdorOBP83a-2-dsRNA-injected insects were also assessed using a wind tunnel (Figure S4).

A fixed setting of 300 ml/min air-flow was used during the assays. Odor molecules pass through the glass tube from the odor source to the other end of the pipe along with the air flow. In each trial, one tested fly was placed in the opposite end of the odor source as the starting point. The time needed for insects to reach the odor source from the starting point was determined. The odor source was made by dissolving the test chemicals into paraffin oil at a final concentration of 10^−2^ v/v. Twenty microliters of the resulting mixture were added onto an absorbent cotton ball, which was used immediately as odor source. The wind tunnel experiment was recorded with 10 individuals of each sex, and performed in triplicates. Both EAG and wind tunnel experiments were performed at 25°C with relative humidity 90%.

### Statistical analysis

Statistical analyses were performed using Prism 6.0 (GraphPad Software, CA, USA). Statistical significance levels were derived through ANOVA and adjusted by a Tukey multiple comparison test.

## Results and discussion

### OBP gene expression levels and changes in insect olfactory behavior

**Figure 1:**
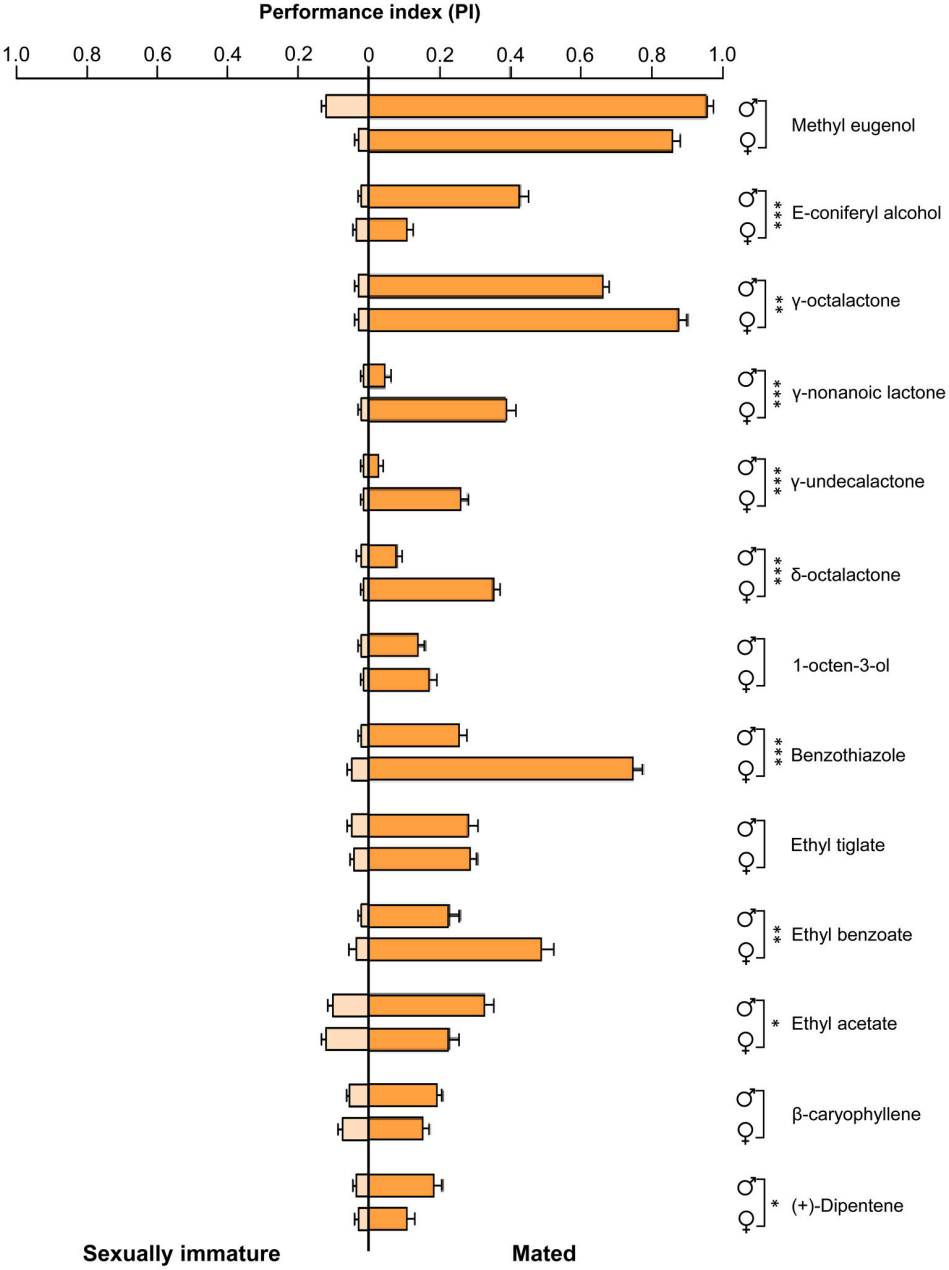
**Olfactory behavior responses of sexually immature and mated males and females of *B. dorsalis* to selected attractant chemicals**. Asterisks indicate significant differences in performance index of males and females (^*^P < 0.05, ^**^P < 0.01, ^***^P < 0.0001, unpaired two-tailed *t*-tests).

**Figure 2:**
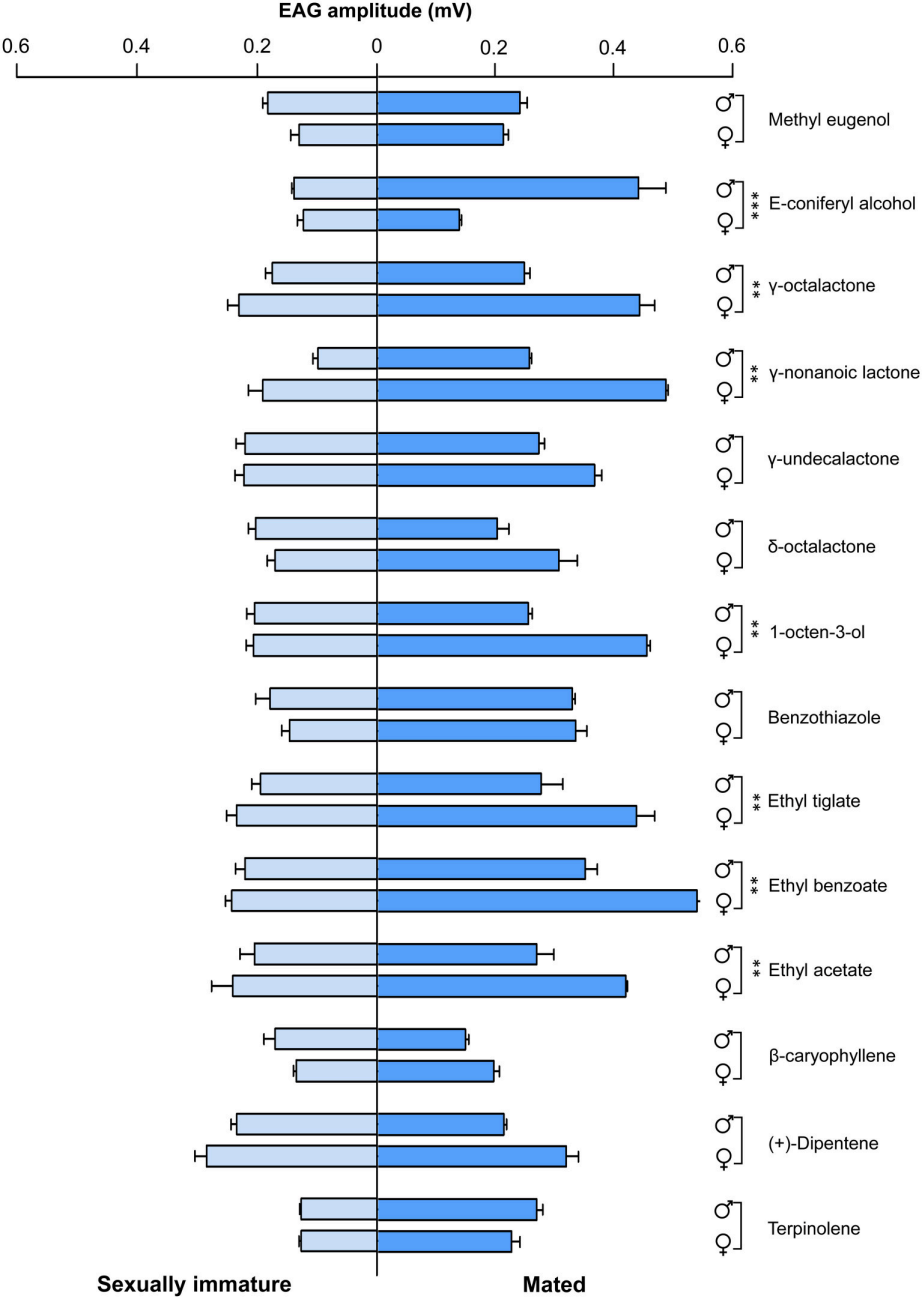
**EAG responses of sexually immature and mated males and females of *B. dorsalis* to selected chemicals**. Asterisks indicate significant differences in EAG responses of males and females (^**^P < 0.01, ^***^P < 0.0001, unpaired two-tailed *t*-tests).

To determine olfactory behavioral changes in different stages of *B. dorsalis*, we used traps with different attractants to measure olfactory reactions in sexually immature and mated individuals from both sexes. We conducted olfactory assays with attractants that have been previously reported effective to *B. dorsalis* adults (Table S1; Chiu, 1990; Hwang et al., 2002; Tan et al., 2010; Hu et al., 2012; Tan and Nishida, 2012; Kamala Jayanthi et al., 2014; Jayanthi et al., 2014; Pagadala Damodaram et al., 2014). We found that immature males and females responded relatively weakly to nearly all tested attractant compounds (left part of Figure 1, Table S7). In contrast, both mated males and females responded strongly to most of the tested attractant compounds (right part of Figure 1). To some chemicals including E-coniferyl alcohol, γ-octalactone, and benzothiazole, mated males and females reacted differently. We also examined EAG responses on the same types of insects with the same set of chemicals. The EAG data exhibited similar differences between immature and mated flies as observed in olfactory behavior except that immature insects responded more strongly to the tested chemicals than what was observed in olfactory response assays (Figure 2). Mated males and females also responded differently to the attractants E-coniferyl alcohol and γ-octalactone, but reacted similarly to benzothiazole.

**Figure 3:**
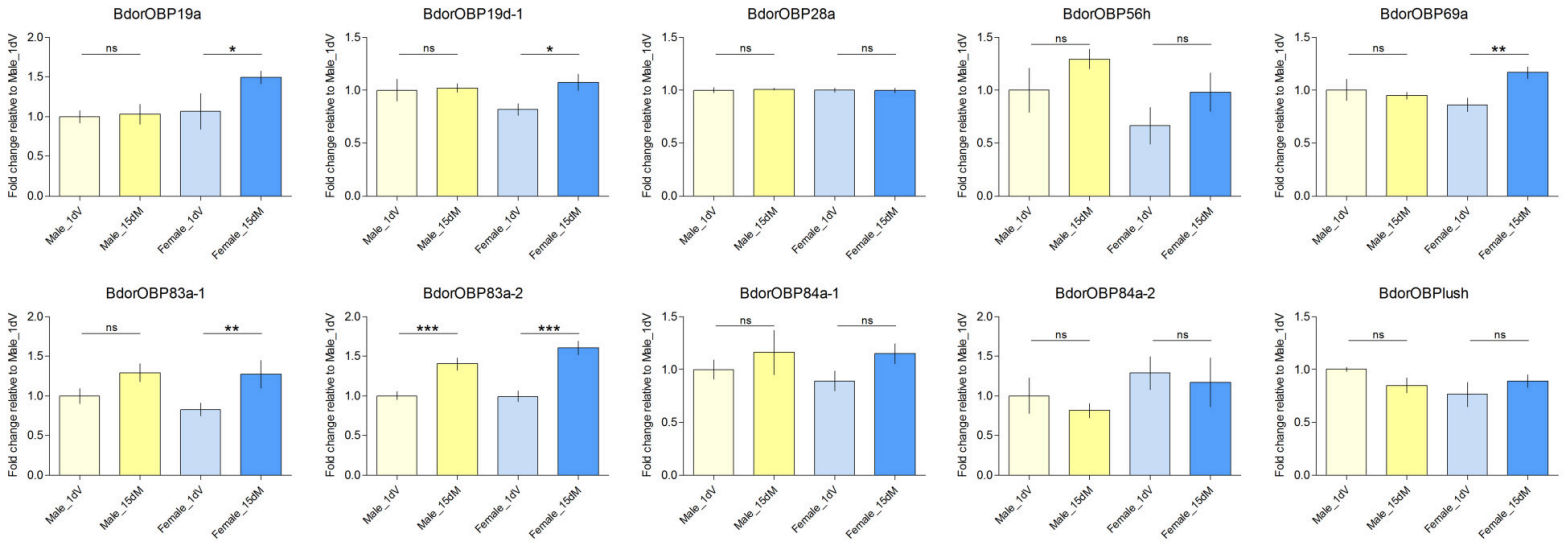
**Transcript abundance of selected OBP genes in the antennae of 1 day immature (1 dV), and 15 days old mated (15 dM) males and females**. Asterisks indicate significant differences in transcript abundances (^*^P < 0.05, ^**^P < 0.01, ^***^P < 0.0001, unpaired two-tailed *t*-tests).

**Figure 4:**
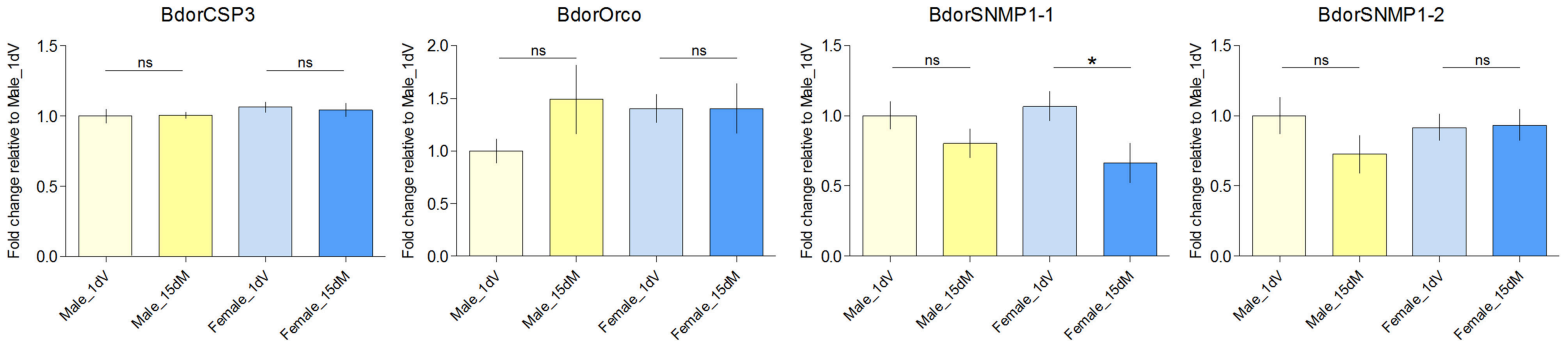
**Transcript abundance of BdorCSP3, BdorOrco, BdorSNMP1-1, BdorSNMP1-2 in the antennae of 1 day immature (1 dV), and 15 days old mated (15 dM) males and females**. Asterisks indicate significant differences in transcript abundances (^*^P < 0.05, unpaired two-tailed *t*-tests).

Previous reports indicate that expression levels of olfactory genes correlate with antennal odorant receptivity in the mosquito *Anopheles gambiae* (Rinker et al., 2013a,b), the tsetse fly *G. m. morsitans* (Liu et al., 2010, 2012), and the beet armyworm *Spodoptera exigua* (Wan et al., 2015). Here, we wanted to investigate whether expression levels of olfactory genes could regulate both behavioral and neuronal responses in *B. dorsalis* at different physiological status. Previously, we determined the expression profiles of individual genes (Wu et al., 2015). The following antenna-specific or antenna-predominant olfaction genes, including 10 OBPs (BdorOBPlush, BdorOBP19a, BdorOBP19d-1, BdorOBP28a, BdorOBP56h, BdorOBP69a, BdorOBP83a-1, BdorOBP83a-2, BdorOBP84a-1), one CSP (BdorCSP3), one ORco (BdorORCO), and two SNMPs (BdorSNMP1-1 and BdorSNMP1-2), are expressed exclusively or predominantly in antennae. At the present study, we further determined if these antenna-specific genes were differentially expressed in different developmental stages of male and female adults. We found that five OBP genes were significantly upregulated in mated females during oviposition compared with immature females, whereas only one was significantly upregulated in mated males (Figure 3). These observations suggested that the specific upregulation of OBPs in mated adults might be involved in changes in olfactory perception. However, CSPs and ORco genes were not significantly upregulated in either mated females or males, and a significant down-regulation of SNMP1-1 in females was observed under our conditions (Figure 4). Expression of genes encoding antenna-specific chemosensory proteins varies in different insect species. In *G. m. morsitans*, antenna-specific CSPs (GmmCSP2), the orthologs of BdorCSP3, are up-regulated during starvation in female adults, and are thought to be linked to olfactory perception of hosts (Liu et al., 2012). In *A. gambiae*, a subset of OBP genes are upregulated post a blood feeding whereas most other chemosensory genes are not affected or even down-regulated (Rinker et al., 2013a); and such changes following blood feeding are coincident with a switch from host-seeking to oviposition behaviors. These observations suggest that different mechanisms likely exist for chemosensory perception in different insects under different conditions.

Olfactory behavior of most insects displays remarkable phase changes associated with different physiological status, such as sexually immature and mated adults. Typically, mated male fruit flies are strongly attracted to and compulsively feed on methyl eugenol, but sexually immature males showed a weak behavioral response to methyl eugenol (Mitchell et al., 1985; Tan and Nishida, 2012). In addition, various fruit volatiles attract gravid females, which lay their eggs in host fruits (Cornelius et al., 2000; Hwang et al., 2002; Siderhurst and Jang, 2006; Jayanthi et al., 2012; Kamala Jayanthi et al., 2014; Pagadala Damodaram et al., 2014). On the contrary, sexually immature females showed limited responses to food-type attractants, such as fermenting sugars, hydrolyzed protein, and yeast. In this study, the expression level of BdorOBP83a-2 along with other antenna-specific OBPs correlated with increased olfactory sensitivity, indicating that specific OBPs were likely responsible for detection of specific attractants.

### Binding assays of OBPs to attractant semiochemicals

**Figure 5:**
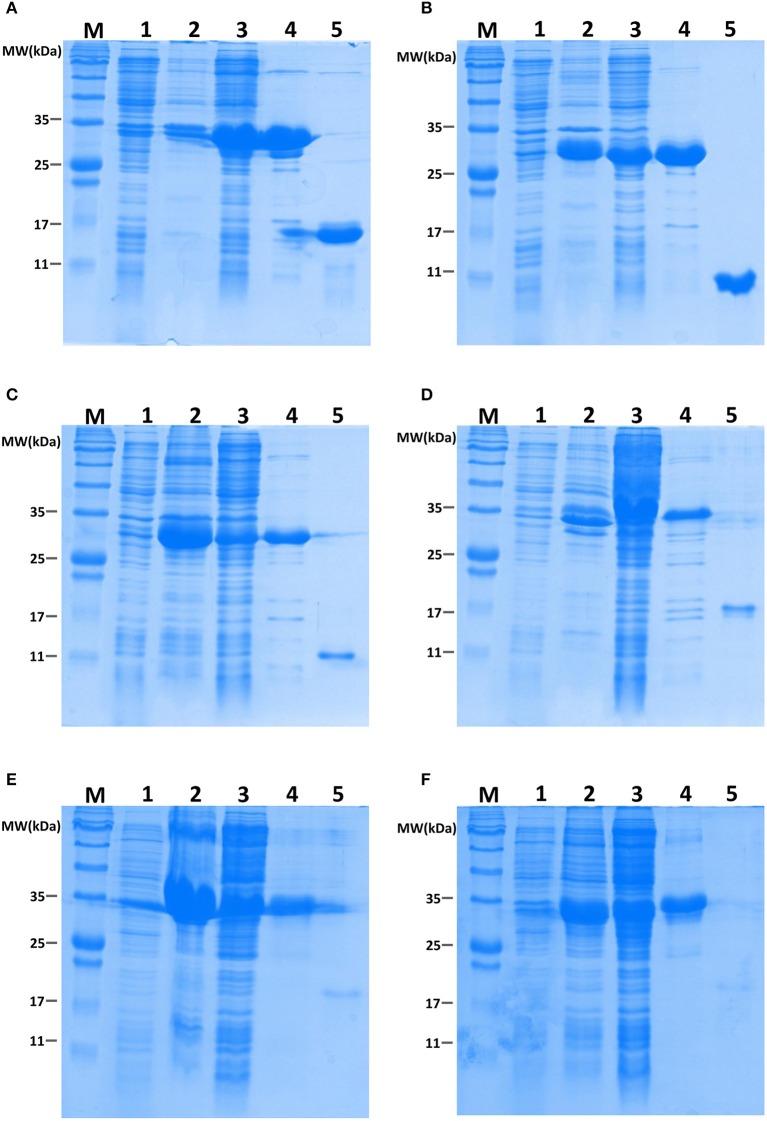
**Recombinant BdorOBPs and a BdorCSP analyzed on SDS-PAGE**. M, Molecular weight marker; 1, Total protein extract from non-induced pET32a transformed BL21 (DE3) cells; 2, Protein extract from isolated inclusion body of pET32a transformed cells; 3, Supernatant of ultrasonated pET32a transformed cells; 4, Proteins purified through Ni-NTA columns; 5, Purified proteins with His-tag cleaved using recombinant enterrokinase; **(A)**, BdorCSP3; **(B)**, BdorOBP56h; **(C)**, BdorOBP83a-1; **(D)**, BdorOBP83a-2; **(E)**, BdorOBP84a-1; **(F)**, BdorOBP84a-2.

**Figure 6:**
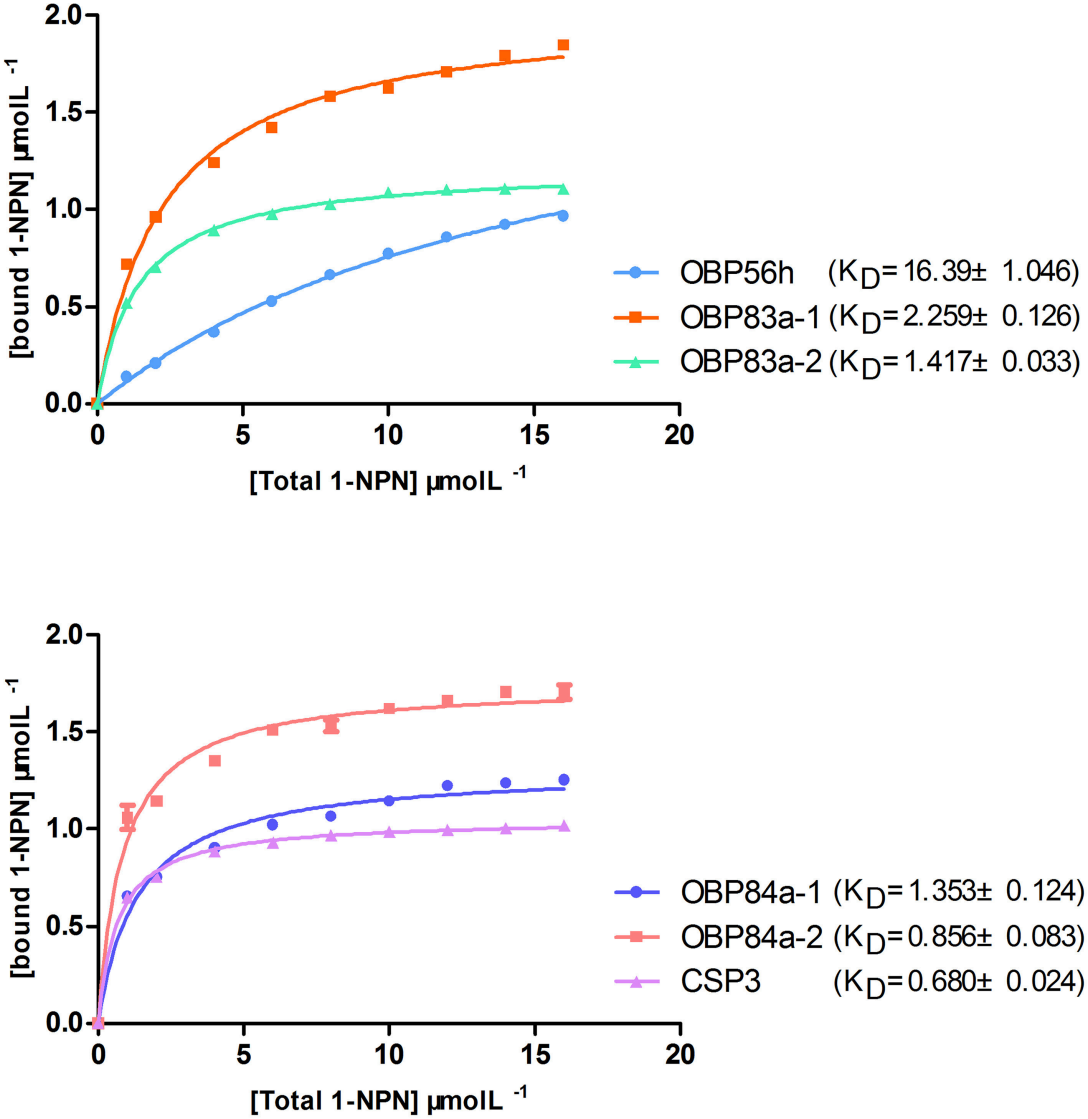
**Binding assays of recombinant OBPs and a CSP with the fluorescent probe 1-NPN**. Data are means of three independent experiments.

**Figure 7:**
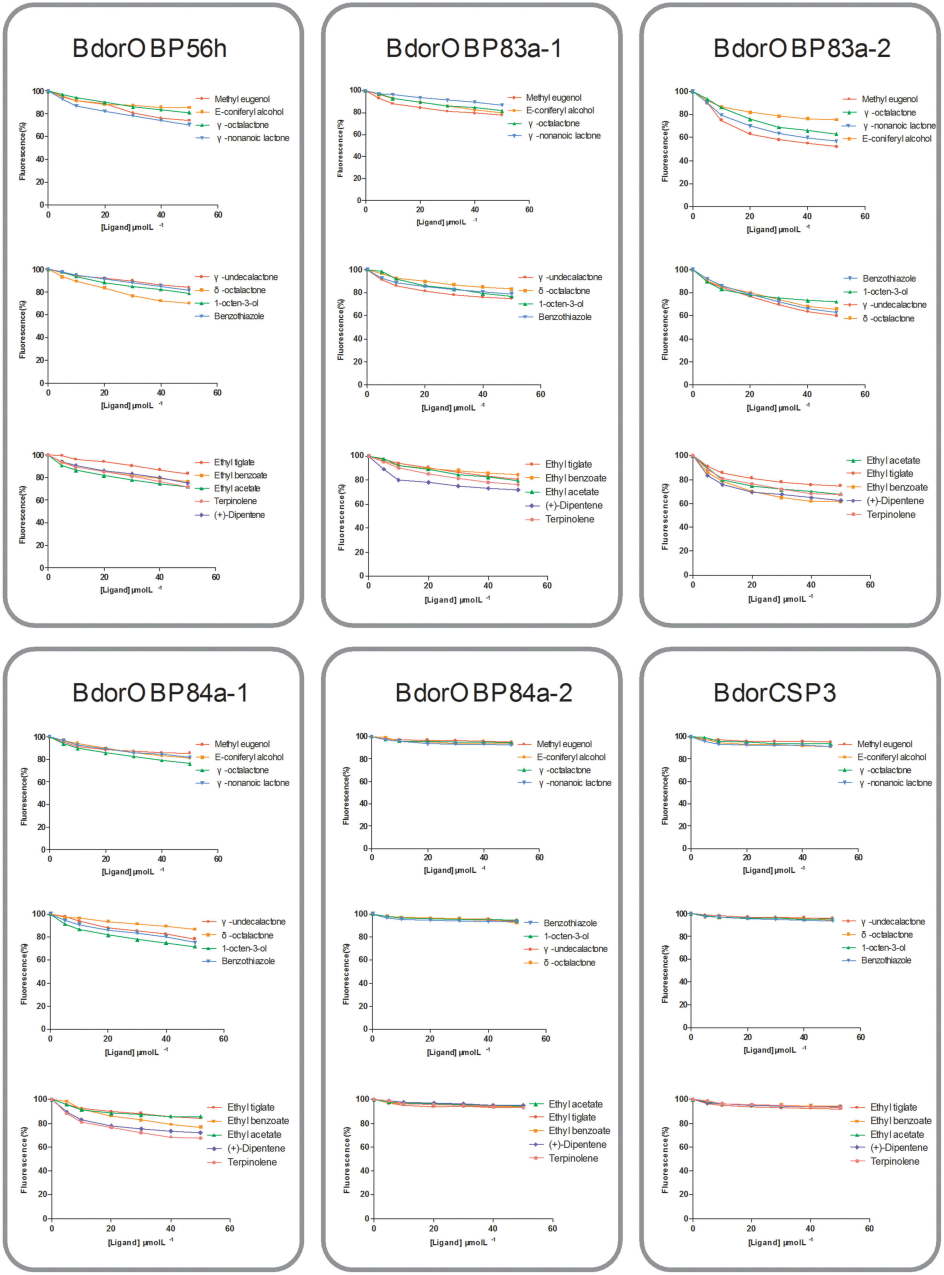
**Binding assays of recombinant OBPs and a CSP with selected ligands**. Data are means of two independent experiments.

To determine the roles of specific OBPs in recognition of attractants in *B. dorsalis*, recombinant proteins of selected antenna-abundant OBPs and CSPs were obtained using a prokaryotic expression system (Figure 5). Purified OBPs and CSPs were used to determine binding affinity of each protein via fluorescence competitive binding assays. Thirteen pure attractant compounds were tested against each recombinant protein. Six antenna-rich proteins (five OBPs and one CSP) exhibited high affinity to the fluorescent probe 1-NPN (Figure 6). Except for BdorCSP3 and BdorOBP84a-2, all remaining OBPs could bind to the tested compounds (Figure 6; Table S8). The binding inability of BdorCSP3 and BdorOBP84a-2 might suggest that other compounds could be ligands for these two proteins. Alternatively they might be involved in functions other than odor detection. Among the analyzed proteins, BdorOBP83a-1 and BdorOBP83a-2 showed higher affinity than the remaining OBPs to all tested attractant chemicals, with BdorOBP83a-2 being the highest (Table S8). For BdorOBP83a-2, attractants methyl eugenol and esters exhibited the lowest dissociation constants (Figure 7; Table S8). The binding affinity decreased in order for attractants methyl eugenol, γ-nonanoic lactone, δ-octalactone, γ-undecalactone, and γ-octalactone. Overall, we observed lower binding affinity with our recombinant proteins and tested attractants. As shown in Figure 7, the highest binding affinity was with BdorOBP83a-2, which reached approximately 40% of displacement in competitive binding assays. In other studies, much higher affinity has been reported. For example, competitive binding assays with a lepidopteran pheromone-binding protein can reach nearly 100% displacement (Hooper et al., 2009; Gu et al., 2013), and competitive binding assays with an aphid OBP (OBP7) can reach about 80% displacement with the alarm pheromone (E)-ss-farnesene (Sun et al., 2012). The biological significance of the observed lower affinity with *B. doralis* OBPs here remains to be determined. It could be due to diverse binding mechanisms between different functional OBPs and the corresponding compounds. Alternatively, BdorOBP83a-2 may interact with high affinity with other odor chemicals not yet identified.

Possible 3-dimensional structures of five OBPs and one CSP were predicted through molecular simulation (Figure S5), and the interactions between each of the six proteins and thirteen compounds were tested through docking (Table S9). Each protein displayed distinct spectra of binding affinity with the tested chemicals. Among them, BdorOBP83a-1 and BdorOBP83a-2 showed the highest affinity on average. Our overall simulation and docking data were in agreement with binding assays.

Phylogenetic analysis revealed that two OBP83a homologs, BdorOBP83a-1 and BdorOBP83a-2, clustered together with orthologous OBPs from other Dipterans (Figure S6), consistent with exceptional conservation in function among OBP83a orthologs from different species (Figure S7). OBP83a orthologs, which were exclusively expressed in antennae, have been reported to play crucial roles in olfactory perception, such as in starved females in host seeking of *G. m. morsitans* (Liu et al., 2010), and pheromone components detection in *C. capitata* (Siciliano et al., 2014a,b). These results correspond well to its specific tissue distribution within antennae which could give a functional implication that BdorOBP83a-1 and BdorOBP83a-2 would share a relatively conserved physiological function with its orthologs.

### Roles of BdorOBP83a-2 in attractant perception

**Figure 8:**
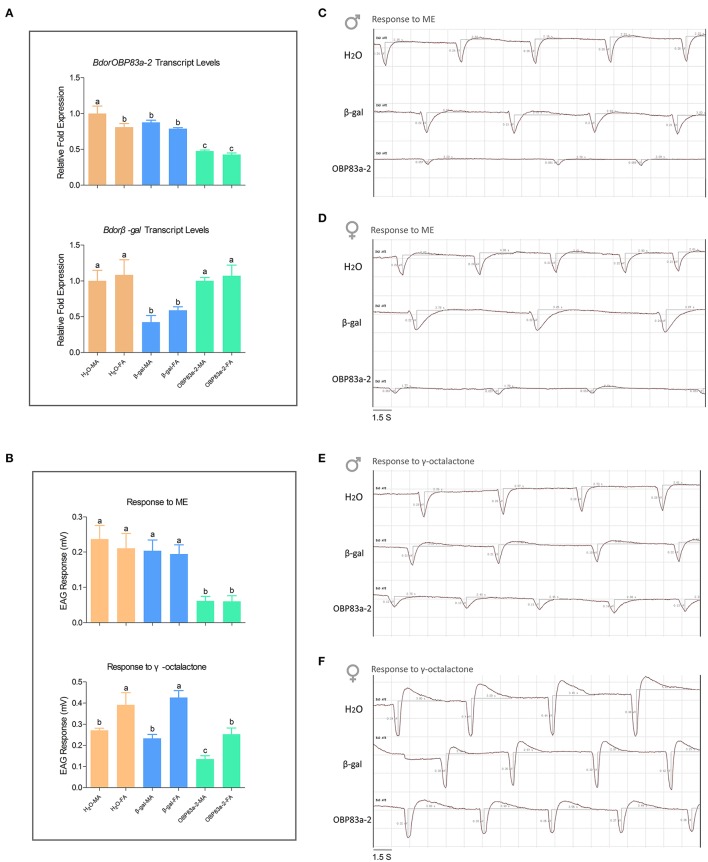
**Effect of RNAi treatments on electrophysiological responses to methyl eugenol and γ-octalactone**. **(A)** Upper part: BdorOBP83a-2 transcript levels in water-treated (orange), β-galactosidase-dsRNA-injected (blue), and BdorOBP83a-2-dsRNA-injected the oriental fruit fly (lime); lower part: Bdorβ-gal transcript levels in water-treated (orange), β-galactosidase-dsRNA-injected (blue), and BdorOBP83a-2-dsRNA-injected the oriental fruit fly (lime); **(B)** upper part: EAG responses to methyl eugenol in water-treated (orange), β-galactosidase-dsRNA-injected (blue), and BdorOBP83a-2-dsRNA-injected; lower part: EAG responses to γ-octalactone in water-treated (orange), β-galactosidase-dsRNA-injected (blue), and BdorOBP83a-2-dsRNA-injected; EAG traces recorded from antennae of water-treated, β-galactosidase-dsRNA-injected, and BdorOBP83a-2-dsRNA-injected male and female challenged with methyl eugenol **(C,D)** and γ-octalactone (**E,F**). MA, male antennae; FA, female antennae. Different lowercase letters above each bar denote significant differences between samples (*p* < 0.05).

**Figure 9:**
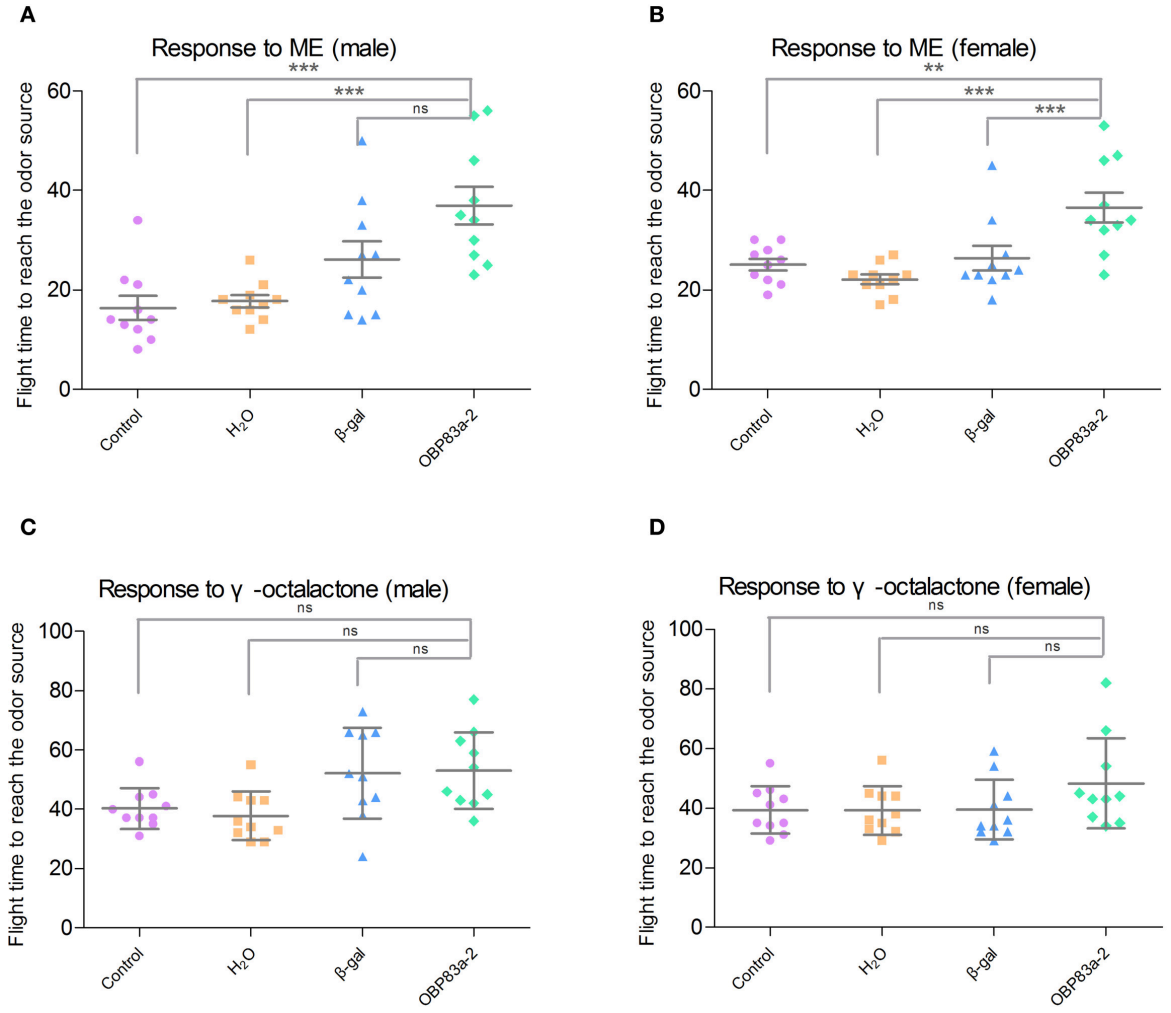
**Effect of RNAi treatments on olfactory behavior to the attractants methyl eugenol and γ-octalactone**. Olfactory behavior to methyl eugenol in non-treated (purple), water-treated (orange), β-galactosidase-dsRNA-injected (blue), and BdorOBP83a-2-dsRNA-injected (green) in males **(A)** and females **(B)**; and to γ-octalactone in males **(C)** and females **(D)** (^**^P < 0.01, ^***^P < 0.0001).

Since BdorOBP83a-2 was upregulated in both mated males and females, and exhibited the highest ligand-binding affinity, we further analyzed its potential role in olfactory perception via RNAi. Microinjection of BdorOBP83a-2 dsRNA inhibited approximately 50% expression of the gene in antennae based on qPCR analysis (Figure 8A). Males and females with BdorOBP83a-2 knocked down were examined for possible impact on olfactory behavior changes. As shown in Figures 8B(upper part),C,D, knockdown of BdorOBP83a-2 resulted in 60–70% reduction of EAG response activity to methyl eugenol. Similarly, knockdown of BdorOBP83a-2 resulted in approximately 40% reduction of EAG response activity to γ-octalatone (lower part of Figures 8B,E,F). In comparison, knockdown of olfactory-unrelated control genes resulted in no significant changes in EAG response activities. Males and females with BdorOBP83a-2 knocked down were also used for olfactory behavior analysis. Flight time to reach the odor source for insects with BdorOBP83a-2 knocked down increased 30–50% with methyl eugenol as attractant. Knockdown of control genes resulted in no apparent changes in flight time (Figures 9A,B). Our data strongly suggest that BdorOBP83a-2 contributes to the changes observed in chemosensory and behavioral function. Our observation is consistent with reports that knockdown of a single OBP gene led to a significant decrease in the sensitivity of *C. quinquefasciatus* and *A. gambiae* adults to major oviposition attractants (Biessmann et al., 2010; Pelletier et al., 2010a). Mutations in a single OBP gene can also cause significant changes in odorant perception and courtship behavior of Drosophila adults (Xu et al., 2005; Laughlin et al., 2008).

## Conclusions

We have comparatively analyzed olfactory attraction and EAG responses to semiochemicals in sexually immature and mated males and females. The expression of antenna-predominant OBPs was upregulated in the mated flies, and this increase may be related to the need for an increased ability to detect some key volatiles. Our ligand-binding assays demonstrated that OBP83a homologs exhibited the highest affinity to the attractant semiochemicals. Reduction in BdorOBP83a-2 transcript abundance led to a decrease in neuronal responses to representative attractants as well as behavioral responses. Together, these results suggest that BdorOBP83a-2 is likely to participate in mediating responses of *B. dorsalis* adults to attractant semiochemicals.

## Author contributions

ZW and XZ designed the experiments. ZW and HZ performed the experiments. ZW and JL contributed reagents/materials/gene identification. ZW analyzed the data. ZW and XZ wrote and revised the paper.

### Conflict of interest statement

The authors declare that the research was conducted in the absence of any commercial or financial relationships that could be construed as a potential conflict of interest.

## Supplementary material

Table S1 **Detailed information of semiochemicals. IUPAC nomenclature, CAS number (CAS #), Behavioral output, Odor resource, Chemical structure, and References are shown**.

Table S2 **Primers for heterologous expression of OBP and CSP proteins**.

Table S3 **Three-dimensional modeling of proteins using SWISS-MODEL**.

Table S4 **Three-dimensional modeling of proteins using Phyre2**.

Table S5 **Primers used for producing constructs for BdorOBP83a-2 and Bdorβ-gal genes and for PCR amplification of templates for dsRNA synthesis**.

Table S6 **Primers used for qRT-PCR to determine transcript levels of BdorOBP83a-2 and Bdorβ-gal genes**.

Table S7 **Performance index (PI) of response to different compounds in sexual immaturity and sexual maturity**.

Table S8 **Binding characteristics of four recombinant BdorOBPs with selected semiochemicals**.

Table S9 **Outputs of *in silico* docking analyses of selected semiochemicals with putative OBPs and a CSP through the “Docking Server”**.

Figure S1 **Schematic presentation of olfactory trap assays**.

Figure S2 **Alignments of BdorOBP83a-1, BdorOBP83a-2, BdorCSP3, BdorOBP84a-1, BdorOBP84a-2, and BdorOBP56h with the template protein**.

Figure S3 ***Ex vivo* degradation assay of dsRNA fragments using hemolymph**. H_2_O: H_2_O+dsRNA; 0–3 h: hemolymph plasma+dsRNA

Figure S4 **The device used for olfactory behavior assays (A), its dimensions (B) and sketches (C)**.

Figure S5 **Three-dimensional modeling of putative proteins BdorOBP56h, BdorOBP83a-1, BdorOBP83a-2, BdorOBP84a-1, BdorOBP83a-2, and BdorCSP3**.

Figure S6 **Phylogenetic relationship of OBPs from *B. dorsalis* and other dipterans**. Bars indicate branch lengths in proportion to amino acid substitutions per site. Bdor, *Bactrocera dorsalis*; Csty, *Calliphora stygia*; Dmel, *Drosophila melanogaster*; Gmm, *Glossina morsitans morsitans*; Mdom, *Musca domestica*; Rpom, *Rhagoletis pomonella*; Rsua, *Rhagoletis suavis*.

Figure S7 **An amino acid alignment of BdorOBP83a-2 with orthologs from other dipterans**. Signal peptides were removed from all amino acid sequences. All conserved cysteine residues are displayed under the alignment.
