# Supplementary material for: BdorOBP83a-2 Mediates Responses of the Oriental Fruit Fly to Semiochemicals
Source: Front Physiol. 2016 Oct 5;7:452. doi: 10.3389/fphys.2016.00452 (PMC5050210; doi:10.3389/fphys.2016.00452)
Supplement: Table S1 — Detailed information of semiochemicals. IUPAC nomenclature, CAS number (CAS #), Behavioral output, Odor resource, Chemical structure, and References are shown. [file Table1.DOCX]

Table S1. Detailed information of semiochemicals. IUPAC nomenclature, CAS number (CAS #), Behavioral output, Odor resource, Chemical structure, and References are shown.

| IUPAC Name | CAS No. | Behavioral output | Resource | Chemical structure | Refs. |
| --- | --- | --- | --- | --- | --- |
| Methyl eugenol | 93-15-2 | Male attractant | Non-Host | 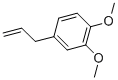 | (1) |
| E-coniferyl alcohol | 458-35-5 | Pheromone | Male | 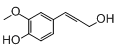 | (2) |
| γ-octalactone | 104-50-7 | Oviposition attractant | Host(mango-Alphonso) | 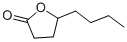 | (3) |
| γ-nonanoic lactone | 104-61-0 | γ-Octalactone analogous |  | 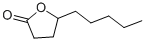 |  |
| γ-undecalactone | 104-67-6 | γ-Octalactone analogous |  | 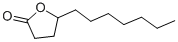 |  |
| δ-octalactone | 698-76-0 | γ-Octalactone analogous |  | 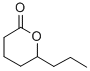 |  |
| 1-octen-3-ol | 3391-86-4 | Oviposition attractant | Host(mango- Totapuri) | 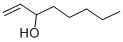 | (4) |
| Ethyl tiglate | 5837-78-5 | Oviposition attractant | Host(mango- Totapuri) | 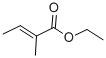 | (4) |
| Benzothiazole | 95-16-9 | Oviposition attractant | Host(mango- Totapuri) | 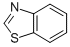 | (4) |
| Ethyl benzoate | 93-89-0 | Oviposition attractant |  | 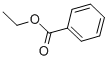 | (5) |
| Ethyl acetate | 141-78-6 | Oviposition attractant | Host(guava) | 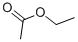 | (6) |
| β-caryophyllene | 87-44-5 | Oviposition attractant | Host(guava) | 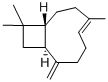 | (6) |
| (+)-Dipentene | 5989-27-5 | Oviposition attractant | Host(citrus) | 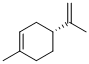 | (7) |

**References:**

1. Tan, K.H., and Nishida, R. (2012). Methyl eugenol: Its occurrence, distribution, and role in nature, especially in relation to insect behavior and pollination. *J*. *Insect*. *Sci*. 12, 1-74.
2. Tan, K.H., Tokushima, I., Ono, H., and Nishida, R. (2010). Comparison of phenylpropanoid volatiles in male rectal pheromone gland after methyl eugenol consumption, and molecular phylogenetic relationship of four global pest fruit fly species: *Bactrocera invadens*, *B. dorsalis*, *B. correcta*, and *B. zonata*. *Chemoecology*. 21, 25-33.
3. Pagadala Damodaram, K.J., Kempraj, V., Aurade, R.M., Venkataramanappa, R.K., Nandagopal, B., Verghese, A., Bruce, T. (2014). Oviposition site-selection by *Bactrocera dorsalis* is mediated through an innate recognition template tuned to γ-octalactone. *PLoS One*. 23, e85764.
4. Kamala Jayanthi, P.D., Kempraj, V., Aurade, R.M., Venkataramanappa, R.K., Nandagopal, B., Verghese, A., Bruce, T.J. (2014). Specific volatile compounds from mango elicit oviposition in gravid *Bactrocera dorsalis* females. *J*. *Chem*. *Ecol.* 40, 259-266.
5. Chiu, H.T. (1990) Ethyl benzoate: an impact ovipositional attractant of the oriental fruit fly, *Dacus dorsalis* Hendel. *Chinese J. Entomol.* 10: 375-387.
6. Hwang, J.S., Yen, Y.P., Chang, M.C., Liu, C.Y. (2002). Extraction and identification of volatile components of guava fruits and their attraction to oriental fruit fly, *Bactrocera dorsalis* (Hendel). *Plant Protection Bulletin* Taipei 44, 279-302.
7. Hu, L.M., Shen, J.M., Bin, S.Y., Chen, G.F., Lin, B.Q., Lin, J.T. (2012). Chemical constituents of the essential oils from Citrus reticulate and its influence on the oviposition of *Bactrocera dorsalis* (Diptera: Tephritidae). *Journal of Fruit Science*, 29, 630-633.
