# Supplementary material for: BdorOBP83a-2 Mediates Responses of the Oriental Fruit Fly to Semiochemicals
Source: Front Physiol. 2016 Oct 5;7:452. doi: 10.3389/fphys.2016.00452 (PMC5050210; doi:10.3389/fphys.2016.00452)
Supplement: Table S2 — Primers for heterologous expression of OBP and CSP proteins. [file Table2.DOCX]

Table S2. Primers for heterologous expression of OBP and CSP proteins.

| Primer name | Sequence (5′-3′) |
| --- | --- |
| BdorOBP56h_F | GCGCTCGAGTTAGAGATTATTCTGTGT |
| BdorOBP56h_R | GCGCTCGAGTTAGAGATTATTCTGTGT |
| BdorOBP83a-1_F | ATCCATGGCACAGGAACCGCGACGTG |
| BdorOBP83a-1_R | GGCCTCGAGTTATGGTAGGAAATAATG |
| BdorOBP83a-2 F | ACCATGGCGCAAAAGGAGCTGAGACGT |
| BdorOBP83a-2_R | GGCCTCGAGTTAGATCAAGAAATAATGC |
| BdorOBP84a-1_F | GGATCCCATCCTGAAACAGATGCAAC |
| BdorOBP84a-1_R | CTCGAGTCAAACCAACGGCTTATTGC |
| BdorOBP84a-2_F | CCATGGCACAGGATCGCGCCAAGGAT |
| BdorOBP84a-2_R | GCCTCGAGTTAGGCAACAGTATCATTGC |
| BdorCSP3_F | CCCATGGCTGCACCGCATCCACCGAC |
| BdorCSP3_R | GCCTCGAGTCAGGCTTGTGTCTTAGT |

The underlined indicate the restriction sites.
