# Supplementary material for: BdorOBP83a-2 Mediates Responses of the Oriental Fruit Fly to Semiochemicals
Source: Front Physiol. 2016 Oct 5;7:452. doi: 10.3389/fphys.2016.00452 (PMC5050210; doi:10.3389/fphys.2016.00452)
Supplement: Table S3 — Three-dimensional modeling of proteins using SWISS-MODEL. [file Table3.DOCX]

Table S3. Three-dimensional modeling of proteins using SWISS-MODEL.

| Gene name | Accession ID | GMQE | Template | Seq Identity | Species/Description | Refs. |
| --- | --- | --- | --- | --- | --- | --- |
| BdorOBP83a-1 | KP743699 | 0.89 | 3ogn | 63.64% | *C. quinquefasciatus*/Odorant-binding protein | (1) |
| BdorOBP83a-2 | KP743700 | 0.94 | 3ogn | 68.60% | *C. quinquefasciatus* /Odorant-binding protein | (1) |
| BdorCSP3 | KP743661 | 0.67 | 2gvs | 46.60% | *S. gregaria*/chemosensory protein CSP-sg4 | (2) |

GMQE（Global Model Quality Estimation) is a quality estimation which combines properties from the target-template alignment.

**References:**

1. Mao, Y., Xu, X., Xu, W., Ishida, Y., Leal, W.S., Ames, J.B., Clardy, J. (2010). Crystal and solution structures of an odorant-binding protein from the southern house mosquito complexed with an oviposition pheromone. *Proc Natl Acad Sci U S A.* 107, 19102-7.
2. Tomaselli, S., Crescenzi, O., Sanfelice, D., Ab, E., Wechselberger, R., Angeli, S., et al. (2006)*.* Solution structure of a chemosensory protein from the desert locust *Schistocerca gregaria*. *Biochemistry.* 45,10606-13.
