# Supplementary material for: BdorOBP83a-2 Mediates Responses of the Oriental Fruit Fly to Semiochemicals
Source: Front Physiol. 2016 Oct 5;7:452. doi: 10.3389/fphys.2016.00452 (PMC5050210; doi:10.3389/fphys.2016.00452)
Supplement: Table S4 — Three-dimensional modeling of proteins using Phyre2. [file Table4.DOCX]

Table S4. Three-dimensional modeling of proteins using Phyre2.

| Gene name | Accession ID | Template | Confidence | Coverage | Species/Description | Refs. |
| --- | --- | --- | --- | --- | --- | --- |
| BdorOBP56h | KP743696 | 4inw | 99.9% | 100% | *Amyelois transitella* /pheromone-binding protein 1 | （di Luccio et al., 2013） |
| BdorOBP84a-1 | KP743704 | 3q8i | 99.5% | 67% | *A. gambiae*/odorant binding protein 4 | （Davrazou et al., 2011） |
| BdorOBP84a-2 | KP743705 | 3s0b | 99.8% | 69% | *Apis mellifera*/ odorant binding protein 14 | （Spinelli et al., 2012） |

**References**

1. di Luccio, E., Ishida, Y., Leal, W.S., Wilson, D.K. (2013). Crystallographic observation of pH-induced conformational changes in the *Amyelois transitella* pheromone-binding protein AtraPBP1. *PLoS One*, 8, e53840.
2. Davrazou, F., Dong, E., Murphy, E.J., Johnson, H.T., Jones, D.N. (2011).New insights into the mechanism of odorant detection by the malaria-transmitting mosquito *Anopheles gambiae*. *J*. *Biol*. *Chem*. 286, 34175-83.
3. Spinelli, S., Lagarde, A., Iovinella, I., Legrand, P., Tegoni, M., Pelosi, P., Cambillau, C. (2012). Crystal structure of Apis mellifera OBP14, a C-minus odorant-binding protein, and its complexes with odorant molecules. *Insect Biochem Mol Biol*, 42, 41-50.
