# Supplementary material for: BdorOBP83a-2 Mediates Responses of the Oriental Fruit Fly to Semiochemicals
Source: Front Physiol. 2016 Oct 5;7:452. doi: 10.3389/fphys.2016.00452 (PMC5050210; doi:10.3389/fphys.2016.00452)
Supplement: Table S5 — Primers used for producing constructs for BdorOBP83a-2 and Bdorβ-gal genes and for PCR amplification of templates for dsRNA synthesis. [file Table5.DOCX]

Table S5. Primers used for producing constructs for BdorOBP83a-2 and Bdorβ-gal genes and for PCR amplification of templates for dsRNA synthesis.

| Primers | Primer sequence (5′-3′) | PCR amplification products (bp) |
| --- | --- | --- |
| BdorOBP83a-2-F | GAGCTGAGACGTGATGAAACC | 343 bp |
| BdorOBP83a-2-R | CTTCCTTCCAGCAGCGATG |  |
| dsBdorOBP83a-2-F | T7- GAGCTGAGACGTGATGAAACC | 343 bp |
| dsBdorOBP83a-2-R | T7- CTTCCTTCCAGCAGCGATG |  |
| Bdorβ-gal-F | ATGGATGAGGCGGGTAATGT | 394bp |
| Bdorβ-gal-R | GCACCGACAAGATACCGTTC |  |
| dsBdorβ-gal-F | T7-ATGGATGAGGCGGGTAATGT | 394bp |
| dsBdorβ-gal-R | T7-GCACCGACAAGATACCGTTC |  |

T7：sequencer with promoters T7（5′-TAATACGACTCACTATAGGG-3′)
