# Supplementary material for: BdorOBP83a-2 Mediates Responses of the Oriental Fruit Fly to Semiochemicals
Source: Front Physiol. 2016 Oct 5;7:452. doi: 10.3389/fphys.2016.00452 (PMC5050210; doi:10.3389/fphys.2016.00452)
Supplement: Table S6 — Primers used for qRT-PCR to determine transcript levels of BdorOBP83a-2 and Bdorβ-gal genes. [file Table6.DOCX]

Table S6. Primers used for qRT-PCR to determine transcript levels of BdorOBP83a-2 and Bdorβ-gal genes.

| Primers | Primer sequence (5′-3′) | PCR amplification products (bp) |
| --- | --- | --- |
| BdorOBP83a-2 |  | 200 |
| BdorOBP83a-2-F | CTCCCGAAAGACTCTCCTGG |  |
| BdorOBP83a-2-R | GAACATCCCCATCGCTGAAC |  |
| Bdorβ-gal |  | 210 |
| Bdorβ-gal-F | CGGTTTCTCTACGGCAATGG |  |
| Bdorβ-gal-R | ATCAACTGTCGCATGTACGC |  |
| Bdorα-TUB |  | 184 |
| Bdorα-TUB-F | CGCATTCATGGTTGATAACG |  |
| Bdorα-TUB-R | GGGCACCAAGTTAGTCTGGA |  |
