# Supplementary material for: BdorOBP83a-2 Mediates Responses of the Oriental Fruit Fly to Semiochemicals
Source: Front Physiol. 2016 Oct 5;7:452. doi: 10.3389/fphys.2016.00452 (PMC5050210; doi:10.3389/fphys.2016.00452)
Supplement: Table S7 — Performance index (PI) of response to different compounds in sexual immaturity and sexual maturity. [file Table7.DOCX]

Table S7. Performance index (PI) of response to different compounds in sexual immaturity and sexual maturity.
