# Supplementary material for: BdorOBP83a-2 Mediates Responses of the Oriental Fruit Fly to Semiochemicals
Source: Front Physiol. 2016 Oct 5;7:452. doi: 10.3389/fphys.2016.00452 (PMC5050210; doi:10.3389/fphys.2016.00452)
Supplement: Table S8 — Binding characteristics of four recombinant BdorOBPs with selected semiochemicals. [file Table8.DOCX]

Table S8. Binding characteristics of four recombinant BdorOBPs with selected semiochemicals.

|  | BdorOBP56 | | BdorOBP83a-1 | | BdorOBP83a-2 | | BdorOBP84a-1 | |
| --- | --- | --- | --- | --- | --- | --- | --- | --- |
|  | Kd (μmol /L) | IC50 (μmol /L) | Kd (μmol /L) | IC50 (μmol /L) | Kd (μmol /L) | IC50 (μmol /L) | Kd (μmol /L) | IC50 (μmol /L) |
| Methyl eugenol | 577.57 | 584.94 | 2533.21 | 3612.01 | 33.95 | 50.83 | 79683.13 | 124093.01 |
| E-coniferyl alcohol | 123943.82 | 125525.00 | 2713.50 | 3869.08 | 1105.22 | 1654.78 | 2995.65 | 4665.22 |
| γ-octalactone | 4144.83 | 4197.70 | 5433.02 | 7746.74 | 87.38 | 130.82 | 1259.39 | 1961.29 |
| γ-nonanoic lactone | 497.07 | 503.41 | 85887.86 | 122464.21 | 52.45 | 78.53 | 5266.70 | 8201.99 |
| γ-undecalactone | 20172.35 | 20429.69 | 1129.58 | 1610.62 | 77.94 | 116.69 | 1175.31 | 1830.35 |
| δ-octalactone | 334.21 | 338.48 | 12716.07 | 18131.35 | 54.68 | 81.87 | 78562.70 | 122348.13 |
| 1-octen-3-ol | 1937.64 | 1962.36 | 691.12 | 985.44 | 601.25 | 900.21 | 481.83 | 750.37 |
| Benzothiazole | 5148.20 | 5213.87 | 5388.93 | 7683.87 | 99.41 | 148.84 | 906.78 | 1412.15 |
| Ethyl tiglate | 7412.01 | 7506.56 | 2730.61 | 3893.47 | 1074.11 | 1608.19 | 40806.98 | 63549.97 |
| Ethyl benzoate | 2124.22 | 2151.32 | 43679.72 | 62281.24 | 604.34 | 904.84 | 628.55 | 978.85 |
| Ethyl acetate | 789.61 | 799.69 | 1881.93 | 2683.37 | 212.89 | 318.75 | 79608.10 | 123976.16 |
| (+)-Dipentene | 1360.41 | 1377.76 | 638.20 | 909.98 | 137.30 | 205.58 | 560.22 | 872.46 |
| Terpinolene | 639.78 | 647.94 | 978.89 | 1395.76 | 219.41 | 328.51 | 212.95 | 331.64 |
