# Supplementary figures and images for: BdorOBP83a-2 Mediates Responses of the Oriental Fruit Fly to Semiochemicals
Source: Front Physiol. 2016 Oct 5;7:452. doi: 10.3389/fphys.2016.00452 (PMC5050210; doi:10.3389/fphys.2016.00452)

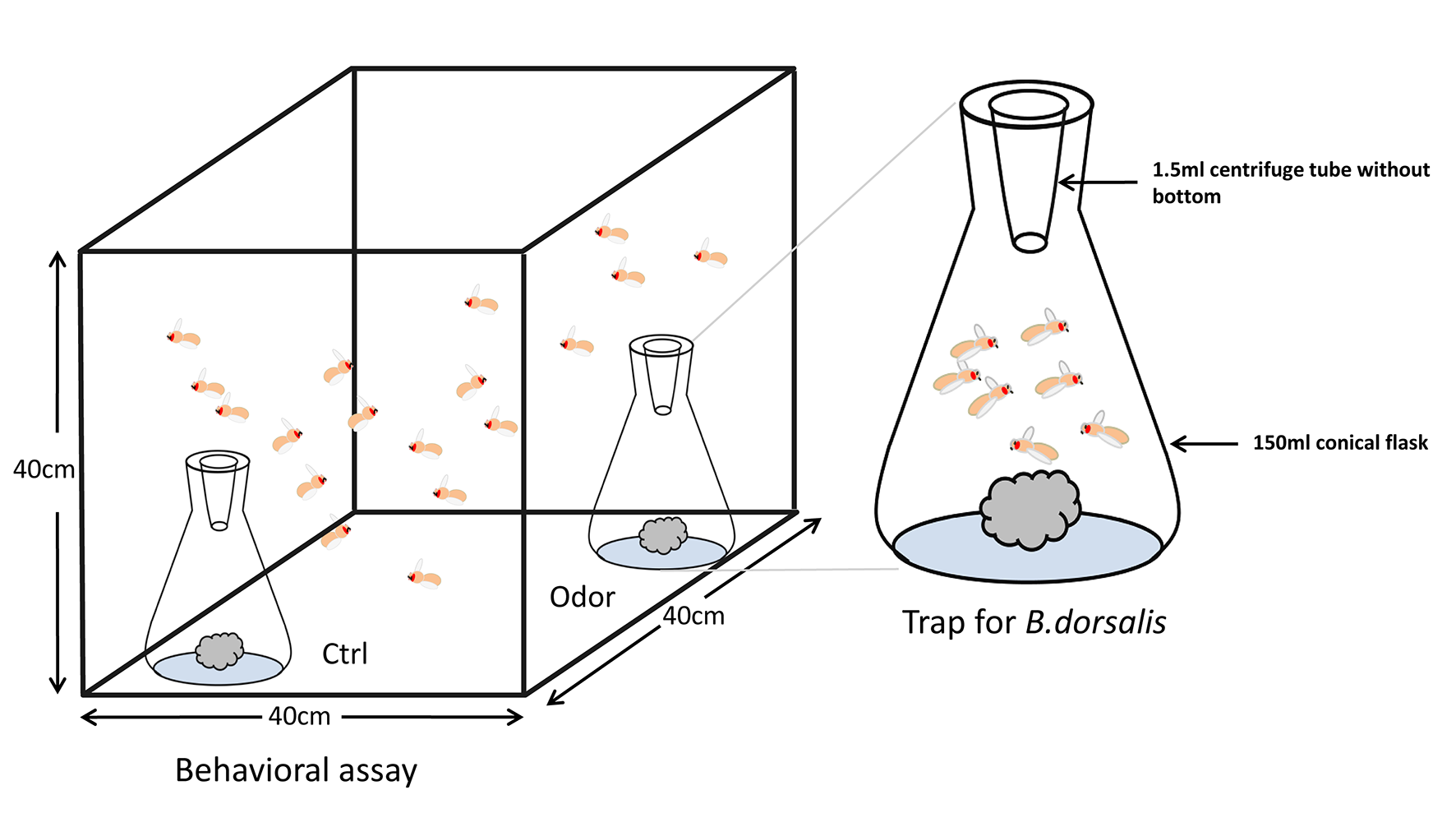

Supplement: Figure S1 — Schematic presentation of olfactory trap assays. [file Image1.TIF]

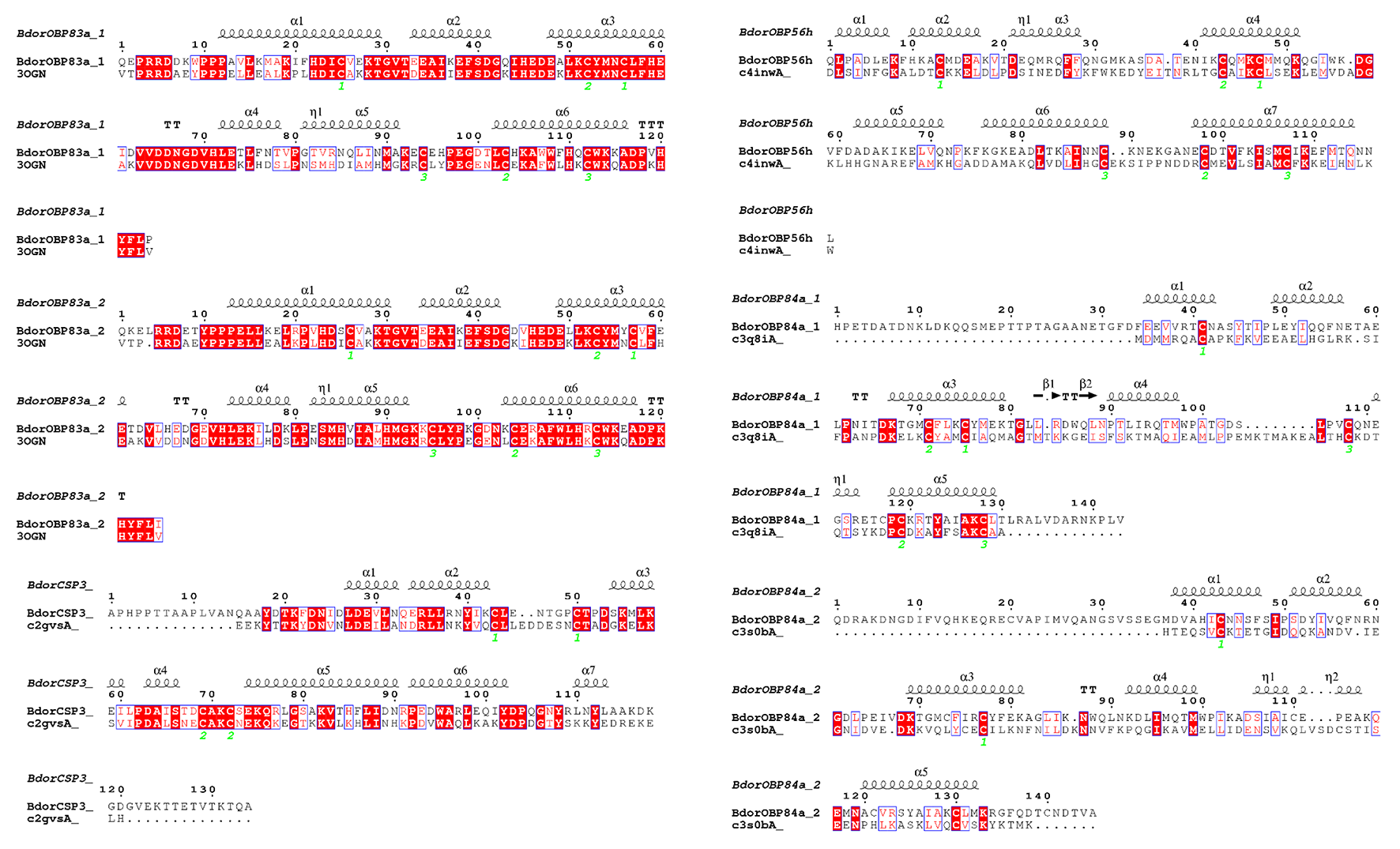

Supplement: Figure S2 — Alignments of BdorOBP83a-1, BdorOBP83a-2, BdorCSP3, BdorOBP84a-1, BdorOBP84a-2, and BdorOBP56h with the template protein. [file Image2.TIF]

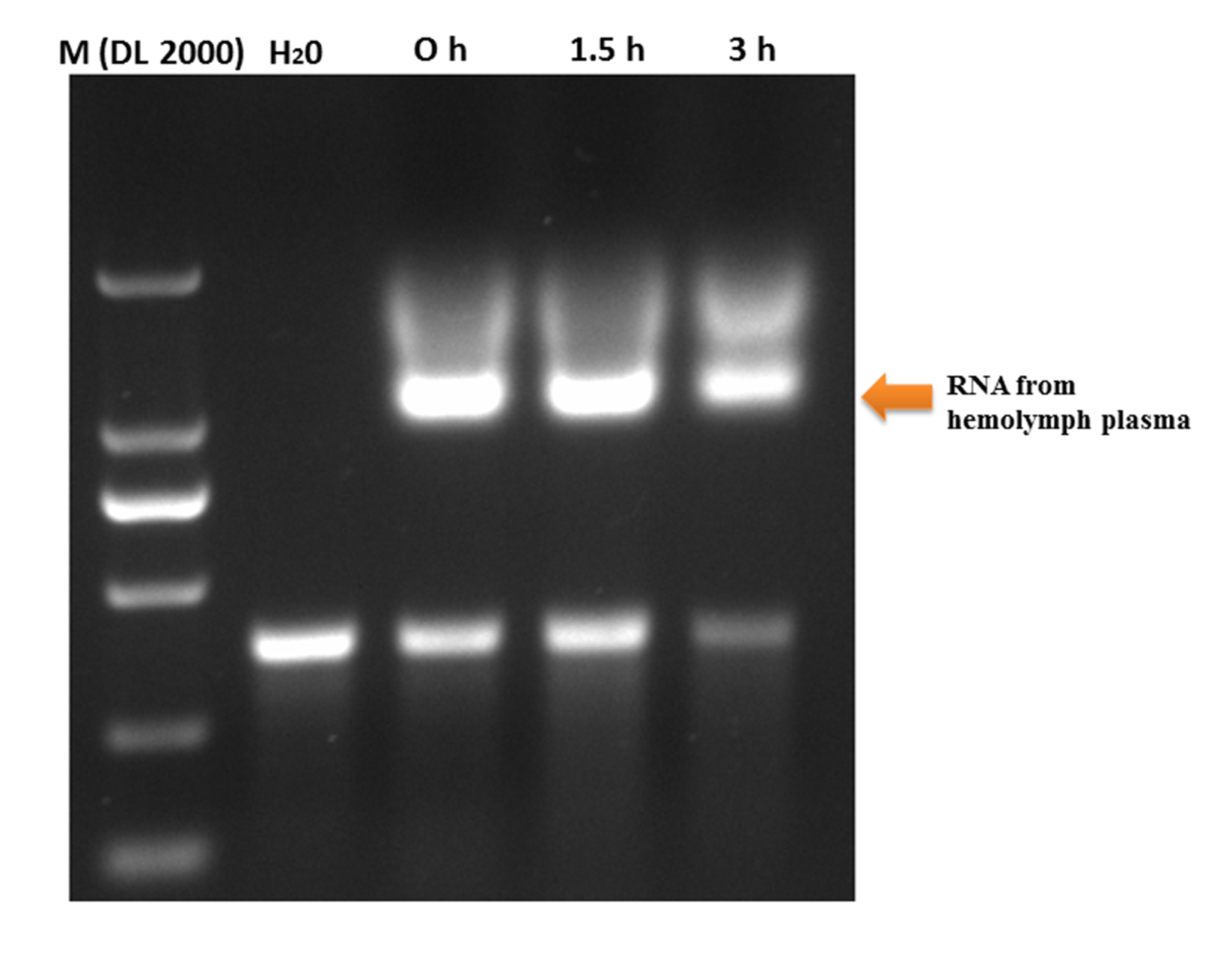

Supplement: Figure S3 — Ex vivo degradation assay of dsRNA fragments using hemolymph. H2O: H2O+dsRNA; 0–3 h: hemolymph plasma+dsRNA [file Image3.TIF]

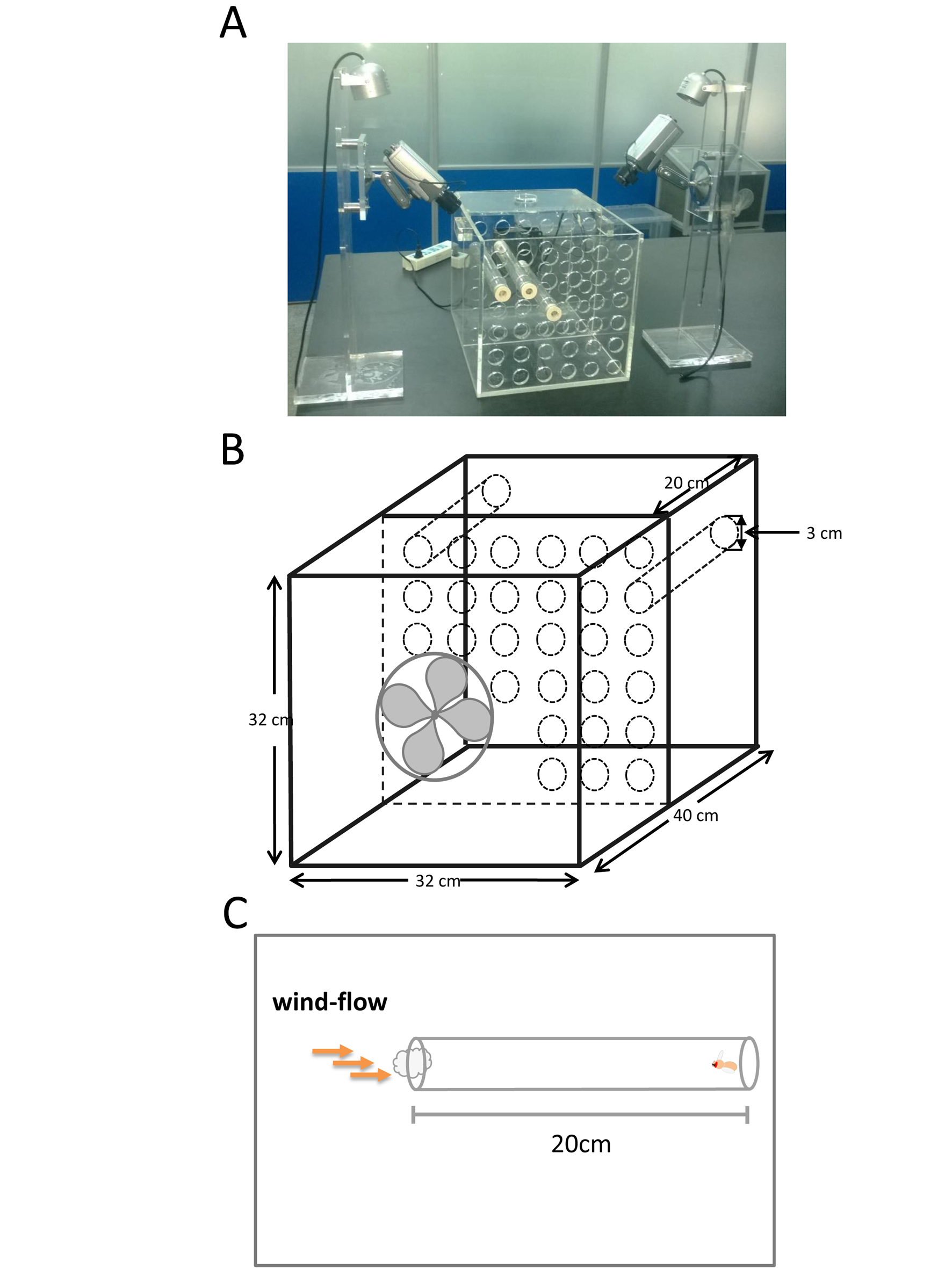

Supplement: Figure S4 — The device used for olfactory behavior assays (A), its dimensions (B) and sketches (C). [file Image4.TIF]

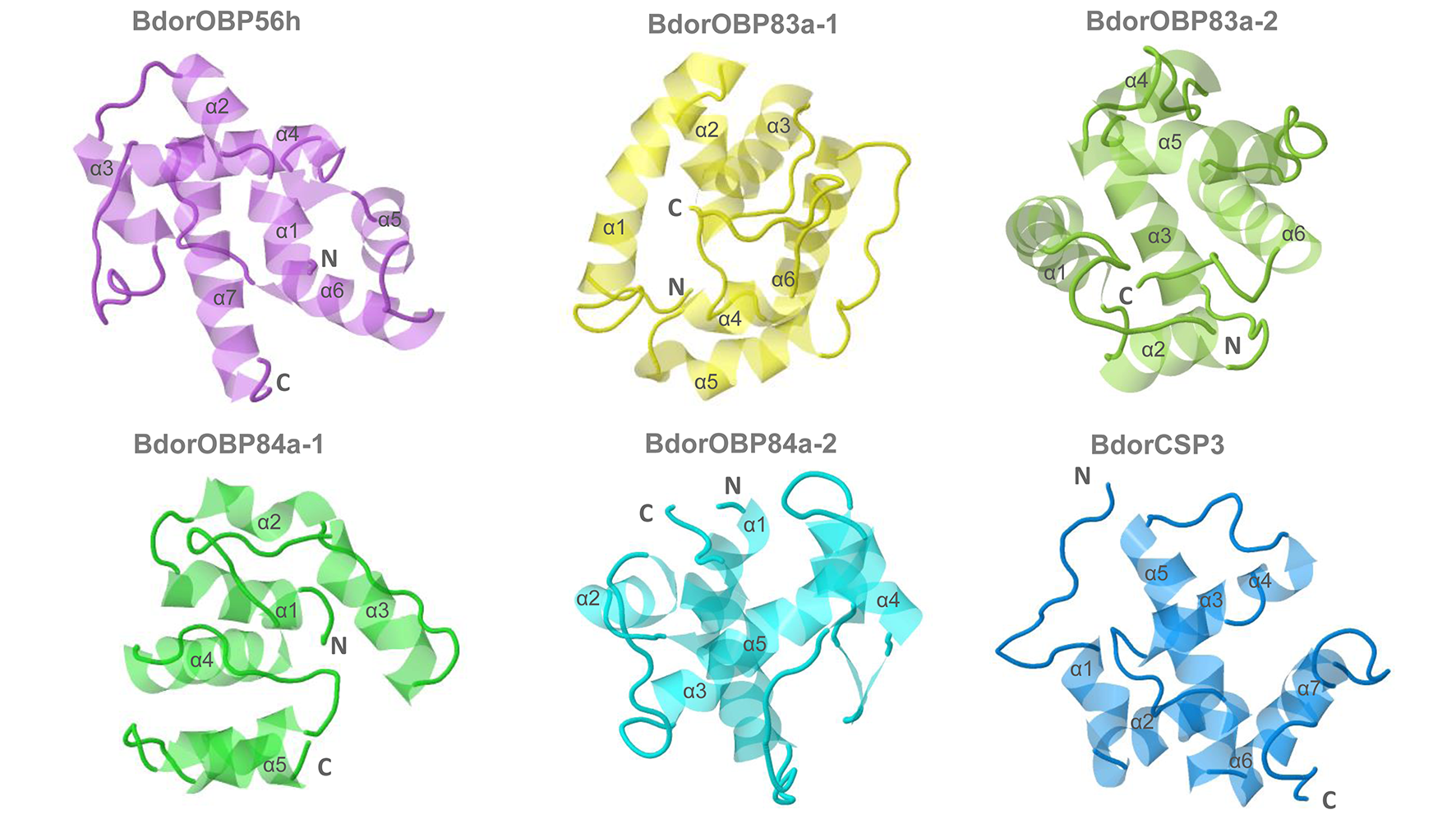

Supplement: Figure S5 — Three-dimensional modeling of putative proteins BdorOBP56h, BdorOBP83a-1, BdorOBP83a-2, BdorOBP84a-1, BdorOBP83a-2, and BdorCSP3. [file Image5.TIF]

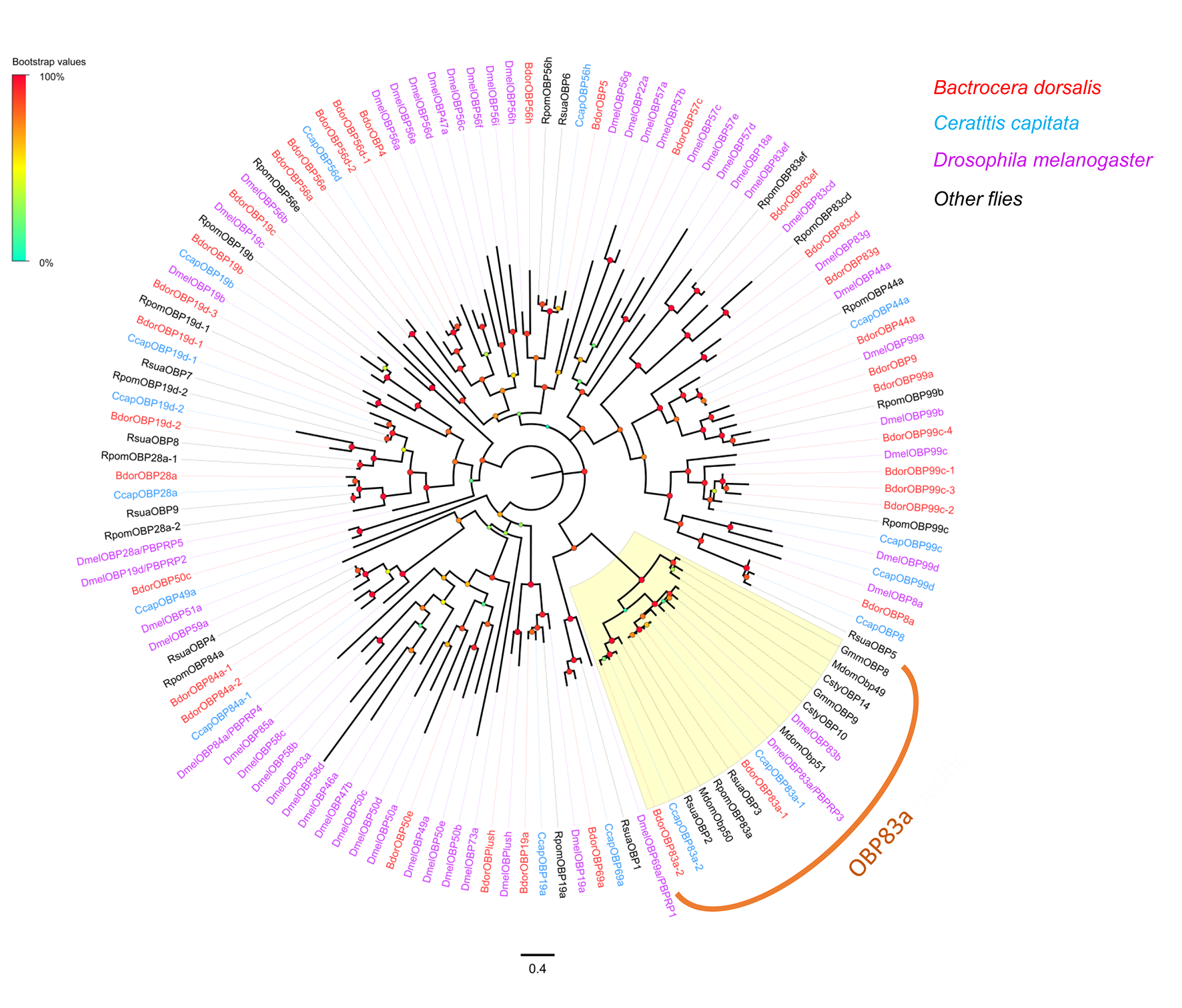

Supplement: Figure S6 — Phylogenetic relationship of OBPs from B. dorsalis and other dipterans. Bars indicate branch lengths in proportion to amino acid substitutions per site. Bdor, Bactrocera dorsalis; Csty, Calliphora stygia; Dmel, Drosophila melanogaster; Gmm, Glossina morsitans morsitans; Mdom, Musca domestica; Rpom, Rhagoletis pomonella; Rsua, Rhagoletis suavis. [file Image6.TIF]

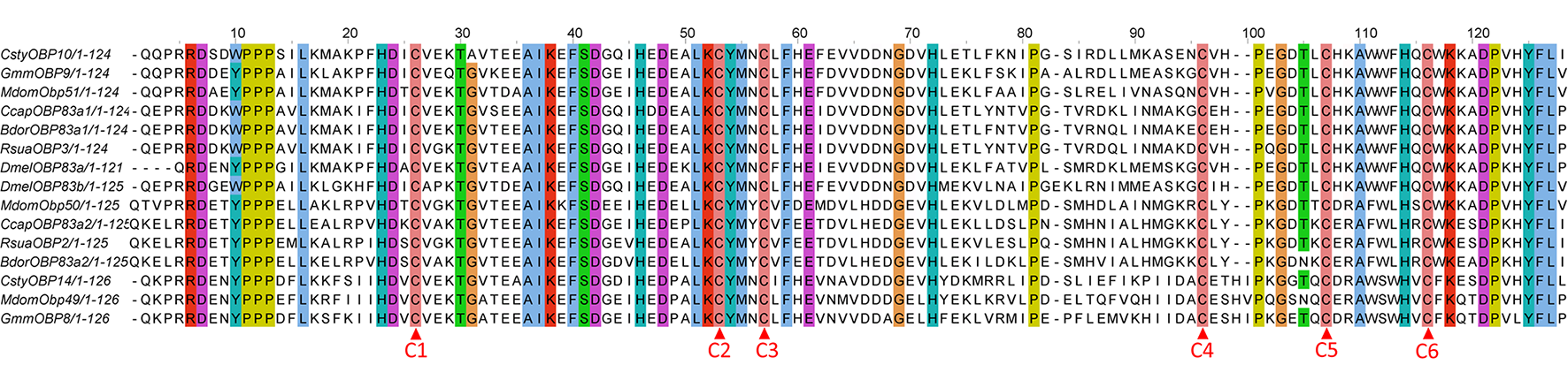

Supplement: Figure S7 — An amino acid alignment of BdorOBP83a-2 with orthologs from other dipterans. Signal peptides were removed from all amino acid sequences. All conserved cysteine residues are displayed under the alignment. [file Image7.TIF]
